# The Future of Medicine

**Published:** 1919-12

**Authors:** 


**The Future of Medicine.** By Sir JAMES MACKENZIE, F.R.S., M.D., etc., Consulting Physician to the London Hospital. Pp. 288. London: Oxford Medical Publications. 1919. Price 8s. 6d.

Two ideas are responsible for a large portion of this volume, (*a*) the deficiencies and failure of medical education, for which the inefficiency of the teachers is mainly responsible, and (*b*) the opportunities and advantages of the general practitioner, who, in consequence of the imperfections of his training, has to go blundering on for many years before he becomes satisfied with his work. The author had no thought of research, as for many years he believed that the great physicians had solved all the problems essential to the recognition of the signs of disease. His eyes were ultimately opened, and the results of his own research work lie patent. Much of this is described in the constructive chapters from page 124, and forms the most useful portion of the book. On previous pages he describes the clinical polygraph, an ingenious elaboration founded on Dudgeon's sphygmograph, on which many useful discoveries have been made, and whereby our improved views as regards irregular heart, auricular fibrillation, the varieties and details of mitral stenosis, and the assessment of the values of symptoms as the ground of prognosis, and other results of the author's research have been established.

It is remarkable to find that the author rather deprecates scientific and laboratory methods, which at the present day are universally encouraged, for he considers that physicians of fifty years ago were able to take a broader outlook than the better trained men of modern days.

There is much to be said in favour of the view that laboratory apparatus gives only a one-sided view of things, whereas the physician should see things from every possible standpoint and form his conclusions accordingly. The general practitioner, doubtless, has many advantages, but these need not depreciate the merits of the consulting physician and the specialist in the way the author does in many of his pages, the more so as he himself has become one of the highly trained group who can afford to be generous to those who may have had fewer advantages.

We think the future of medicine is not so hopeless as is suggested.

